# Male reproductive surgeries and long-term kidney outcomes: a Mendelian randomization and meta-analysis of UK Biobank and FinnGen

**DOI:** 10.1080/0886022X.2026.2696181

**Published:** 2026-08-04

**Authors:** Dianjie Zeng, Lingfei Zhu, Li Shi, Yuxi Wang, Jiachen Liu, Yi Peng, Xiaokun Zhao, Yinhuai Wang, Liya Sun, Haiqing He

**Affiliations:** a Department of Urology, The Second Xiangya Hospital at Central South University, Changsha, Hunan, China; b Xiangya School of Medicine, Central South University, Changsha, Hunan, China; c Department of Nephrology, Xiangya Hospital, Central South University, Changsha, Hunan, China

**Keywords:** Male reproductive surgery, renal outcomes, genetic causal inference, UK biobank, FinnGen, meta-analysis

## Abstract

The long-term renal implications of conditions leading to male reproductive surgeries remain unclear. This study evaluated the potential causal relationships between genetic liability to undergoing these surgeries and kidney diseases. Genetic variants associated with four surgery-related phenotypes—testicular hydrocele surgery, testicular or scrotal surgery, circumcision, and vasectomy—were selected as instrumental variables. Mendelian randomization, meta-analysis, and exploratory mediation analyses were used to assess associations with 20 kidney outcomes. Results showed genetic liability to testicular hydrocele surgery was associated with higher odds of chronic kidney disease, IgA nephropathy, glomerulonephritis, cystic kidney disease, tubulo-interstitial nephritis, and kidney injury, and with lower odds of benign kidney tumors and renal stones. Genetic liability to testicular or scrotal surgery was associated with increased odds of renal colic, gout, malformations of the renal pelvis or ureters, renal stones, and kidney infections. Genetic liability to circumcision was associated with higher odds of congenital obstructive malformations, hypertensive heart/renal disease, malignant kidney neoplasms, and increased kidney volume, and with lower odds of kidney cysts, benign tumors, and chronic renal failure. Genetic liability to vasectomy was linked to reduced odds of pyelonephritis and malignant kidney neoplasms. Mediation analyses suggested partial contributions of immune and metabolic traits. This study provides evidence that genetic liability to undergoing male reproductive system surgeries—reflecting the underlying biological or pathological indications—is associated with distinct patterns of kidney disease susceptibility. Findings suggest that individuals with underlying conditions predisposing them to testicular hydrocele or scrotal surgery may benefit from attention to long-term kidney health.

## Graphical Abstract

**Figure:**
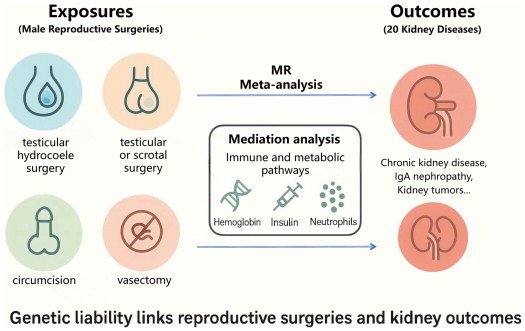

## 1. Introduction

Male reproductive system surgeries, including testicular hydrocele surgery, orchiectomy, vasectomy, and circumcision, are widely performed across the globe to correct anatomical abnormalities or address functional disorders [1–4]. These procedures are generally regarded as safe and effective [5], and their short-term complications—such as infection, pain, hematoma, and sexual dysfunction—have been well characterized [6–9]. However, much less is known about the long-term risk of kidney diseases among individuals who undergo these procedures or among those with underlying biological or developmental conditions that lead to these surgeries.

The male reproductive and urinary systems are closely linked anatomically and physiologically, sharing structures such as the urethra. This interconnection creates opportunities for cross-system effects. For instance, meta-analyses incorporating more than 140 studies collectively reported frequent impairment of reproductive health in patients with chronic kidney disease (CKD) and end-stage renal disease (ESRD) [10,11]. Similarly, reproductive interventions may also influence urinary or renal outcomes. A multicenter cohort study of 69,292 patients demonstrated that prostate cancer patients undergoing orchiectomy had a significantly elevated risk of acute kidney injury (AKI) [12]. These findings highlight the need to investigate whether shared physiological or genetic pathways may contribute to both reproductive surgical indications and renal phenotypes.

Despite such observations, systematic investigations into the long-term renal correlates of male reproductive surgical indications remain scarce. Most existing studies are observational and cannot fully account for confounding by genetics, developmental factors, cultural practices, socioeconomic characteristics, or comorbidities, thereby limiting causal inference. Considering the global burden of kidney disease—affecting more than 750 million people worldwide [13]—and the widespread prevalence of male reproductive surgeries, there is a clear need for a more rigorous analytic approach.

To address this gap, we applied a Mendelian randomization (MR) framework, combined with replication analyses and meta-analysis, to examine whether genetic liability to undergoing four types of male reproductive surgeries—reflecting the underlying biological, developmental, behavioral, or cultural indications for these procedures—is associated with 20 kidney diseases. By leveraging large-scale genomic and epidemiological data, this study aims to clarify the shared genetic architecture linking reproductive surgical indications with renal outcomes and to identify potential pathways for future mechanistic and epidemiological investigation. To our knowledge, this is the first study to systematically evaluate these relationships across multiple male reproductive surgery–related phenotypes and kidney outcomes using both UK Biobank and FinnGen datasets.

## 2. Materials and methods

### 2.1. Study design

**Figure 1.**
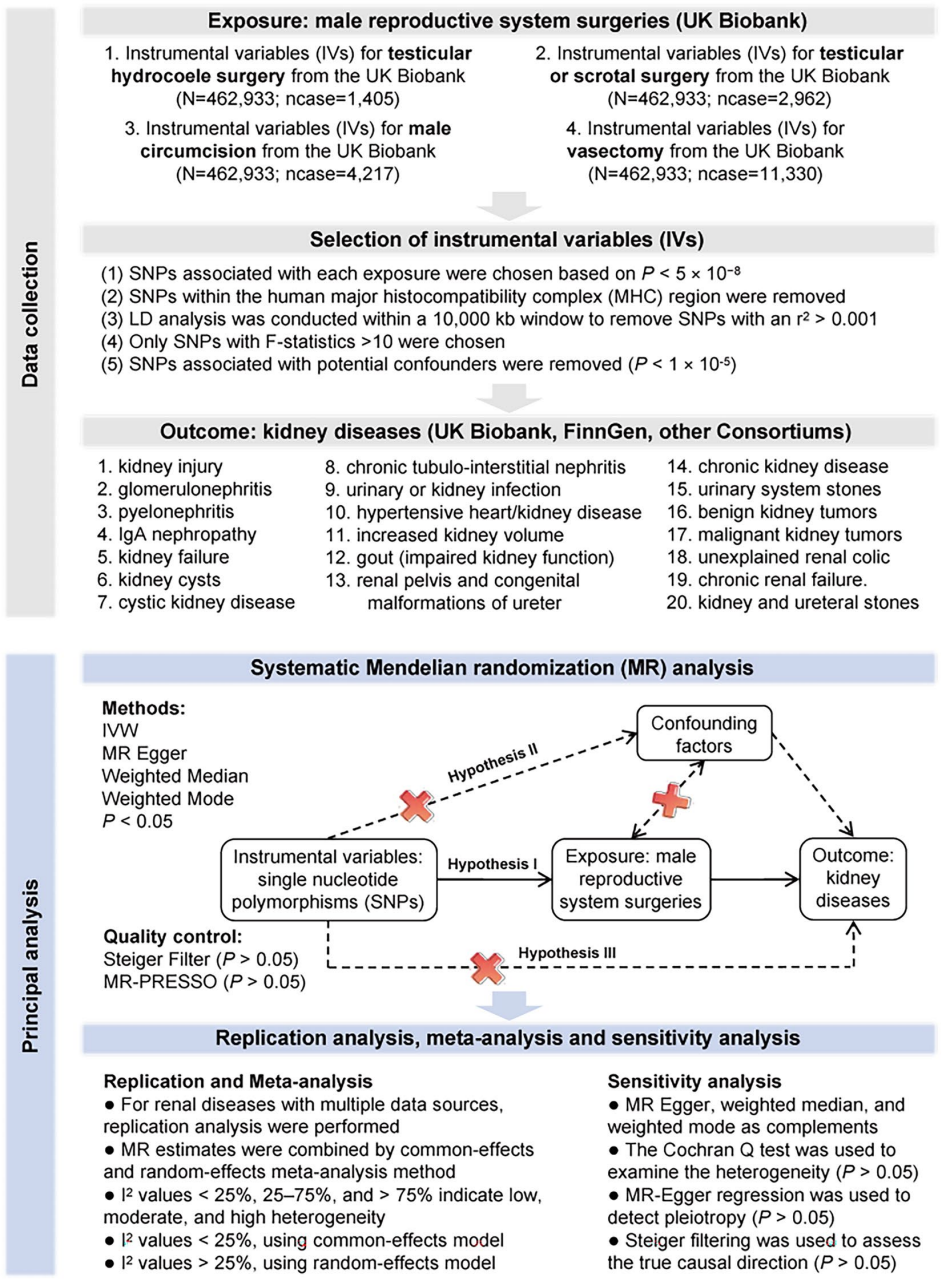
Flowchart of this study.

The overall analytical framework of this study is illustrated in Figure 1. First, based on publicly available genome-wide association study (GWAS) summary statistics, we identified single nucleotide polymorphisms (SNPs) significantly associated with four male reproductive surgery–related phenotypes as instrumental variables (IVs). These SNPs reflect genetic liability to undergoing the procedures, corresponding to the underlying biological, developmental, behavioral, or cultural factors that lead individuals to receive these surgeries. Mendelian randomization (MR) analyses were then performed to investigate the potential causal relationships between this genetic liability and 20 kidney disease outcomes. To increase statistical power and improve the robustness of the findings, replication analyses and meta-analyses were conducted for outcomes with multiple independent GWAS datasets, following methodological principles consistent with recent MR and meta-analytic frameworks applied in renal epidemiology [14,15]. Multiple sensitivity analyses were further applied to evaluate instrument validity and test for potential violations of MR assumptions. This study followed the STROBE-MR reporting guidelines (Supplementary Table S1) [16].

### 2.2. Data sources

This study encompassed four types of male reproductive surgeries: testicular hydrocele surgery (ukb-b-16440), testicular or scrotal surgery (ukb-b-14742), male circumcision (ukb-b-5227), and vasectomy (ukb-b-10167). Summary-level genetic association data for these exposures were obtained from the UK Biobank study (https://www.ukbiobank.ac.uk/), with case numbers ranging from 1,405 (testicular hydrocele surgery) to 11,330 (vasectomy). The UK Biobank is a large-scale prospective cohort study of over 500,000 participants aged 40–69 years recruited across the United Kingdom [17].

For the outcomes, this study included 20 types of kidney diseases: chronic tubulo-interstitial nephritis, glomerulonephritis, pyelonephritis, chronic kidney disease, IgA nephropathy, cystic kidney disease, kidney cysts, kidney injury molecule levels, kidney failure, benign kidney tumors, malignant kidney tumors, unexplained renal colic, gout due to impaired kidney function, kidney and ureteral stones, urinary system stones, urinary tract infection or kidney infection, malformations of renal pelvis and ureters, hypertensive heart/kidney disease, increased kidney volume, and chronic renal failure. Biomarker outcomes, such as kidney injury molecule-1 (KIM-1), were analyzed as continuous traits based on the effect sizes reported in the original GWAS. MR effect estimates for continuous outcomes represent the genetically predicted change in the biomarker level per unit increase in the surgery-coded liability exposure. Summary-level genetic association data for these outcomes were obtained from the FinnGen (https://www.finngen.fi/en), the UK Biobank, and other large-scale GWAS available through the GWAS Catalog (https://www.ebi.ac.uk/gwas/). The FinnGen study is a nationwide research project combining genotype data from Finnish biobanks and digital health record data from national health registries [18]. The GWAS Catalog is an open-access repository that curates published genome-wide association studies and summary statistics [19].

To mitigate potential biases from population stratification, all analyses were restricted to participants of European ancestry. To minimize sample overlap between exposure and outcome datasets, we prioritized outcome GWAS obtained from FinnGen or other independent consortia whenever available. Because all surgery-coded exposures originate from the UK Biobank, complete elimination of overlap is not possible; however, the use of independent outcome datasets substantially reduces this risk. Given that all instruments demonstrated extremely strong strength (*F*-statistics > 1,000), any residual overlap is unlikely to meaningfully bias the MR estimates. Detailed information on each surgery and kidney disease outcome, including GWAS identifiers, year of publication, population, sample sizes, and catalog links, is provided in Table 1.

**Table 1.** Data sources for this study.

Surgeries/Kidney diseases	ID	Year	Population	Sample size	Link
Testicular hydrocele surgery	ukb-b-16440	2018	European	462,933	https://gwas.mrcieu.ac.uk/datasets/ukb-b-16440/
Testicular or scrotal surgery	ukb-b-14742	2018	European	462,933	https://gwas.mrcieu.ac.uk/datasets/ukb-b-14742/
Male circumcision	ukb-b-5227	2018	European	462,933	https://gwas.mrcieu.ac.uk/datasets/ukb-b-5227/
vasectomy	ukb-b-10167	2018	European	462,933	https://gwas.mrcieu.ac.uk/datasets/ukb-b-10167/
Benign neoplasm of kidney	finn-b-CD2_BENIGN_KIDNEY	2021	European	218,792	https://gwas.mrcieu.ac.uk/datasets/finn-b-CD2_BENIGN_KIDNEY/
Benign neoplasm of kidney	finn-b-CD2_BENIGN_KIDNEY_EXALLC	2021	European	180,887	https://gwas.mrcieu.ac.uk/datasets/finn-b-CD2_BENIGN_KIDNEY_EXALLC/
Calculus of kidney and ureter	finn-b-N14_CALCUKIDUR	2021	European	218,414	https://gwas.mrcieu.ac.uk/datasets/finn-b-N14_CALCUKIDUR/
Calculus of kidney and ureter	ukb-a-574	2017	European	337,199	https://gwas.mrcieu.ac.uk/datasets/ukb-a-574/
Chronic kidney disease	ebi-a-GCST003374	2016	European	117,165	https://gwas.mrcieu.ac.uk/datasets/ebi-a-GCST003374/
chronic kidney disease	finn-b-N14_CHRONKIDNEYDIS	2021	European	216,742	https://gwas.mrcieu.ac.uk/datasets/finn-b-N14_CHRONKIDNEYDIS/
Chronic renal failure	ebi-a-GCST90018822	2021	European	482,858	https://gwas.mrcieu.ac.uk/datasets/ebi-a-GCST90018822/
Chronic tubulo-interstitial nephritis	finn-b-N14_CHRONTUBULOINTNEPHRITIS	2021	European	201,648	https://gwas.mrcieu.ac.uk/datasets/finn-b-N14_CHRONTUBULOINTNEPHRITIS/
Cyst of kidney	finn-b-N14_CYSTKID	2021	European	217,924	https://gwas.mrcieu.ac.uk/datasets/finn-b-N14_CYSTKID/
Cystic kidney disease	finn-b-Q17_CYSTIC_KIDNEY_DISEA	2021	European	218,448	https://gwas.mrcieu.ac.uk/datasets/finn-b-Q17_CYSTIC_KIDNEY_DISEA/
Glomerulonephritis	finn-b-GLOMER_NEPHRITIS	2021	European	218,792	https://gwas.mrcieu.ac.uk/datasets/finn-b-GLOMER_NEPHRITIS/
Gout due to impairment of renal function	finn-b-GOUT_KIDNEY	2021	European	215,308	https://gwas.mrcieu.ac.uk/datasets/finn-b-GOUT_KIDNEY/
Hypertensive heart/renal disease	finn-b-I9_HYPTENSHR	2021	European	167,200	https://gwas.mrcieu.ac.uk/datasets/finn-b-I9_HYPTENSHR/
IgA nephropathy	ebi-a-GCST90018866	2021	European	477,784	https://gwas.mrcieu.ac.uk/datasets/ebi-a-GCST90018866/
IgA nephropathy	ieu-a-1081	2010	European	5,957	https://gwas.mrcieu.ac.uk/datasets/ieu-a-1081/
Kidney injury molecule levels	ebi-a-GCST90010147	2020	European	1,301	https://gwas.mrcieu.ac.uk/datasets/ebi-a-GCST90010147/
Kidney injury molecule levels	ebi-a-GCST90012041	2020	European	21,758	https://gwas.mrcieu.ac.uk/datasets/ebi-a-GCST90012041/
Kidney stone/ureter stone/bladder stone	ukb-a-72	2017	European	337,159	https://gwas.mrcieu.ac.uk/datasets/ukb-a-72/
Kidney stone/ureter stone/bladder stone	ukb-b-8297	2018	European	462,933	https://gwas.mrcieu.ac.uk/datasets/ukb-b-8297/
Kidney volume	ebi-a-GCST90016670	2021	European	32,860	https://gwas.mrcieu.ac.uk/datasets/ebi-a-GCST90016670/
malignant neoplasm of kidney	finn-b-C3_KIDNEY_NOTRENALPELVIS	2021	European	218,792	https://gwas.mrcieu.ac.uk/datasets/finn-b-C3_KIDNEY_NOTRENALPELVIS/
Malignant neoplasm of kidney	ukb-b-1316	2018	European	463,010	https://gwas.mrcieu.ac.uk/datasets/ukb-b-1316/
Pyelonephritis	ebi-a-GCST90018909	2021	European	462,487	https://gwas.mrcieu.ac.uk/datasets/ebi-a-GCST90018909/
Renal failure	finn-b-N14_RENFAIL	2021	European	218,792	https://gwas.mrcieu.ac.uk/datasets/finn-b-N14_RENFAIL/
Renal failure	ukb-a-573	2017	European	337,199	https://gwas.mrcieu.ac.uk/datasets/ukb-a-573/
Malformations of renal pelvis and ureters	finn-b-Q17_CONGEN_OBSTRUCTIVE_DEFECTS_RENAL_PELVIS_CONGEN_MALFO_URETER	2021	European	218,235	https://gwas.mrcieu.ac.uk/datasets/finn-b-Q17_CONGEN_OBSTRUCTIVE_DEFECTS_RENAL_PELVIS_CONGEN_MALFO_URETER/
Unspecified renal colic	ukb-b-16313	2018	European	463,010	https://gwas.mrcieu.ac.uk/datasets/ukb-b-16313/
Urinary tract infection or kidney infection	ebi-a-GCST90013890	2021	European	397,867	https://gwas.mrcieu.ac.uk/datasets/ebi-a-GCST90013890/
Urinary tract infection or kidney infection	ebi-a-GCST90013940	2021	European	397,867	https://gwas.mrcieu.ac.uk/datasets/ebi-a-GCST90013940/
Urinary tract infection or kidney infection	ukb-b-7150	2018	European	462,933	https://gwas.mrcieu.ac.uk/datasets/ ukb-b-7150/

### 2.3. Instrumental variable for male reproductive system surgeries

MR relies on three key hypotheses: the genetic instruments are associated with the exposure (relevance); independent of confounders (independence); and influence the outcome only through the exposure (exclusion restriction) [14]. Based on this, SNPs associated with each exposure were selected based on genome-wide significance (*P* < 5e − 8). Subsequently, LD analysis was conducted within a 10,000 kb window to exclude SNPs with an *r*^2^ > 0.001 [20]. The strength of each instrumental variable was assessed using the *F*-statistic, calculated as

$$F=R^{2}(N-K-1)/[K(1-R^{2})],$$

where *R*^2^ represents the proportion of variance in the surgery-coded phenotype explained by the instrument, *N* is the sample size of the exposure GWAS, and *K* is the number of selected SNPs. The proportion of variance in each surgery-coded phenotype explained by the selected SNPs was quantified using the coefficient of determination *R*^2^, computed as

$$R^{2}=2\times \text{EAF}\times (1-\text{EAF})\times \beta^{2},$$

where EAF denotes the effective allele frequency and *β* represents the estimated genetic effect of the SNP on the surgery-coded phenotype. A high *F*-statistic (>10) indicates minimal risk of weak instrument bias, which is crucial in Mendelian randomization studies [21]. Therefore, only SNPs with *F*-statistics exceeding 10 were retained for further analysis (in fact, all *F*-statistics > 1000).

The PhenoScanner GWAS database (http://phenoscanner.medschl.cam.ac.uk) was used to check for associations between IVs and potential confounders (education, smoking, drinking, blood pressure, and socioeconomic status) [22]. SNPs with significant associations (*p* < 1 × 10^−5^) were excluded. It is worth mentioning, although some studies exclude variants within the major histocompatibility complex (MHC) region due to its complex LD structure and pleiotropic functions, we did not apply MHC exclusion in the present analysis. This is because several exposures—particularly male circumcision—have all genome-wide significant variants located within the HLA region. Excluding MHC SNPs would therefore eliminate all valid instruments. To ensure adequate instrument strength, we retained these variants and evaluated potential pleiotropy through comprehensive sensitivity analyses.

Ultimately, six SNPs were retained as IVs for testicular hydrocele surgery, seven for testicular or scrotal surgery, two for male circumcision, and twenty-two for vasectomy (Table 2).

**Table 2.** Instrumental variable for male reproductive system surgeries.

Exposure	SNP	chr	Effect	Other	EAF	*F*	*R* ^2^	Steiger
Testicular hydrocele surgery (ukb-b-16440)	rs6542590	2	A	C	0.55	1605	0.003	True
	rs2215961	2	G	C	0.72	1273	0.003	True
	rs673373	3	C	T	0.49	1245	0.003	True
	rs4568280	4	C	T	0.74	1189	0.003	True
	rs9650064	8	A	G	0.6	1186	0.003	True
	rs2046892	16	A	G	0.46	1159	0.002	True
Testicular/scrotal surgery (ukb-b-14742)	rs1265168	1	G	T	0.17	1205	0.003	True
	rs10071255	5	T	A	0.21	1159	0.003	True
	rs293107	5	C	T	0.51	1535	0.003	True
	rs4702258	5	T	C	0.46	1266	0.003	True
	rs2148342	6	A	G	0.28	1462	0.003	True
	rs7799234	7	C	T	0.62	1188	0.002	True
	rs11103652	9	T	C	0.15	1157	0.002	True
Male circumcision (ukb-b-5227)	rs9275154	6	T	C	0.48	4225	0.009	True
	rs9277621	6	A	G	0.11	2328	0.005	True
Vasectomy (ukb-b-10167)	rs2815752	1	A	G	0.6	1266	0.003	True
	rs138938221	1	A	G	0.06	1372	0.003	True
	rs13404873	2	A	G	0.09	1198	0.003	True
	rs17040885	2	G	T	0.07	1157	0.003	True
	rs12488619	3	G	A	0.92	1222	0.003	True
	rs74396234	3	T	G	0.05	1282	0.003	True
	rs115968765	5	T	C	0.26	1169	0.003	True
	rs7732189	5	G	A	0.47	1169	0.003	True
	rs80107723	5	A	G	0.11	1383	0.003	True
	rs6913664	6	T	C	0.47	1240	0.003	True
	rs849431	7	G	A	0.14	1173	0.003	True
	rs10104544	8	A	G	0.37	1378	0.003	True
	rs7825887	8	G	A	0.37	1166	0.003	True
	rs56687477	8	A	G	0.14	1232	0.003	True
	rs11140385	9	G	A	0.11	1450	0.003	True
	rs7476866	10	C	A	0.32	1354	0.003	True
	rs1367531	11	C	T	0.22	1448	0.003	True
	rs61247097	12	T	G	0.07	1383	0.003	True
	rs17112255	14	T	C	0.12	1364	0.003	True
	rs892061	19	A	G	0.2	1411	0.003	True
	rs6509803	19	A	C	0.81	1211	0.003	True
	rs237732	20	C	T	0.29	1349	0.002	True

### 2.4. Mendelian randomization, meta-analysis and sensitivity analysis

Before conducting MR analyses, exposure and outcome datasets were harmonized using standard procedures implemented in the TwoSampleMR workflow. This included aligning effect alleles, removing ambiguous palindromic SNPs, filtering variants with inconsistent allele frequencies, and ensuring that effect directions were comparable across datasets. The inverse variance weighted (IVW) method was used as the primary MR approach to estimate the potential causal associations between genetic liability to undergoing each surgery-coded phenotype and kidney disease outcomes. Supplementary MR methods, including MR-Egger, weighted median, and weighted mode, were applied to provide estimates that are more robust to different patterns of horizontal pleiotropy. For renal diseases with multiple data sources, we performed replication analysis. Then, MR estimates for each outcome from different sources were combined by the common-effects or random-effects meta-analysis method. The *I*^2^ statistic was calculated to assess the heterogeneity of each outcome from different data sources, with *I*^2^ values < 25%, 25–75%, and > 75% indicating low, moderate, and high heterogeneity, respectively. Choose the appropriate model based on the *I*^2^ statistic and the *P*-value of Cochrans’ *Q* test. For low or moderate heterogeneity (*p* > 0.05), common-effects models were used; for high heterogeneity (*p* < 0.05), random-effects models were used.

To account for multiple comparisons across the large number of exposure–outcome tests, we additionally calculated false discovery rate (FDR)–adjusted *p* values using the Benjamini–Hochberg method. Results that remained significant after FDR correction were highlighted in the Supplementary Materials, whereas associations that did not pass correction were interpreted as exploratory. Sensitivity analyses were performed to test and ensure the robustness of the MR analysis. The Cochrans’ *Q* test was used to examine the heterogeneity of SNPs’ estimates in each MR association. MR-Egger regression was used to detect horizontal pleiotropy using its embedded intercept test and provide estimates corrected for pleiotropic effects [23]. *p* < 0.05 was considered indicative of heterogeneity or pleiotropy. The MR-PRESSO test was applied to detect and remove outlier SNPs that might bias causal estimates (SNPs with *p* < 0.05 were removed). Steiger filtering was conducted to check whether the MR analysis estimates assessed the true causal direction. SNPs with *p* < 0.05 or reversed causal direction (i.e., R^2^_exposure < R^2^_outcome) were removed. All analyses were performed with the TwoSampleMR, MRPRESSO, and meta packages in R software.

### 2.5. Mediation analysis

In addition to the primary MR analyses, we conducted an exploratory mediation analysis to investigate whether genetic liability to undergoing male reproductive system surgeries may be associated with kidney diseases through intermediate biological traits. Because the exposures represent surgery-coded phenotypes, the mediation framework reflects potential pathways linked to the underlying biological, developmental, behavioral, or immunogenetic factors that predispose individuals to these procedures, rather than effects of the surgical interventions themselves. Candidate mediators were selected based on their established biological relevance to kidney disease and their potential involvement in pathways that might also relate to the underlying indications for surgery. These included: (1) clinical indicators such as blood pressure, lipid traits, and kidney injury biomarkers; (2) inflammatory and immune markers, including cytokines and HLA-related traits; and (3) metabolic factors such as fasting insulin and serum albumin levels.

The mediation analysis followed a two-step MR framework. First, we assessed whether genetic liability to each surgery-coded phenotype was associated with a given mediator. Second, we evaluated whether the mediator was associated with kidney disease outcomes in a separate MR analysis. A putative indirect pathway was considered only when both steps demonstrated significant associations (*p* < 0.05), recognizing that such findings represent exploratory, hypothesis-generating signals rather than definitive mechanistic mediation. To evaluate the robustness of these analyses, we applied the same sensitivity procedures used in the primary MR analysis, including Cochran’s *Q* test for heterogeneity, MR-Egger regression for detecting unbalanced horizontal pleiotropy, and MR-PRESSO for identifying outlier variants. Steiger filtering was used to ensure the correct direction of association between exposure, mediator, and outcome (i.e., R^2^_exposure > R^2^_outcome). All mediation analyses were implemented using the same harmonization procedures, MR framework, and software packages as the primary analyses.

### 2.6. Data permission

All GWAS summary statistics used in this study were publicly available, sourced from the UK Biobank, FinnGen, and other large GWAS consortia (*via* the GWAS Catalog), and no special permissions or individual-level data access were required. All statistical analyses were performed in R (version 4.3.2). Mendelian randomization analyses were conducted using the TwoSampleMR package (version 0.5.6), and horizontal pleiotropy was assessed using the MRPRESSO package (version 1.0). Meta-analyses were performed using the meta package (version 6.5-0). Full reproducibility is ensured by specifying all core functions used, including extract_instruments(), harmonise_data(), mr(), and mr_presso().

## 3. Results

### 3.1. Testicular hydrocele surgery and kidney diseases

**Figure 2.**
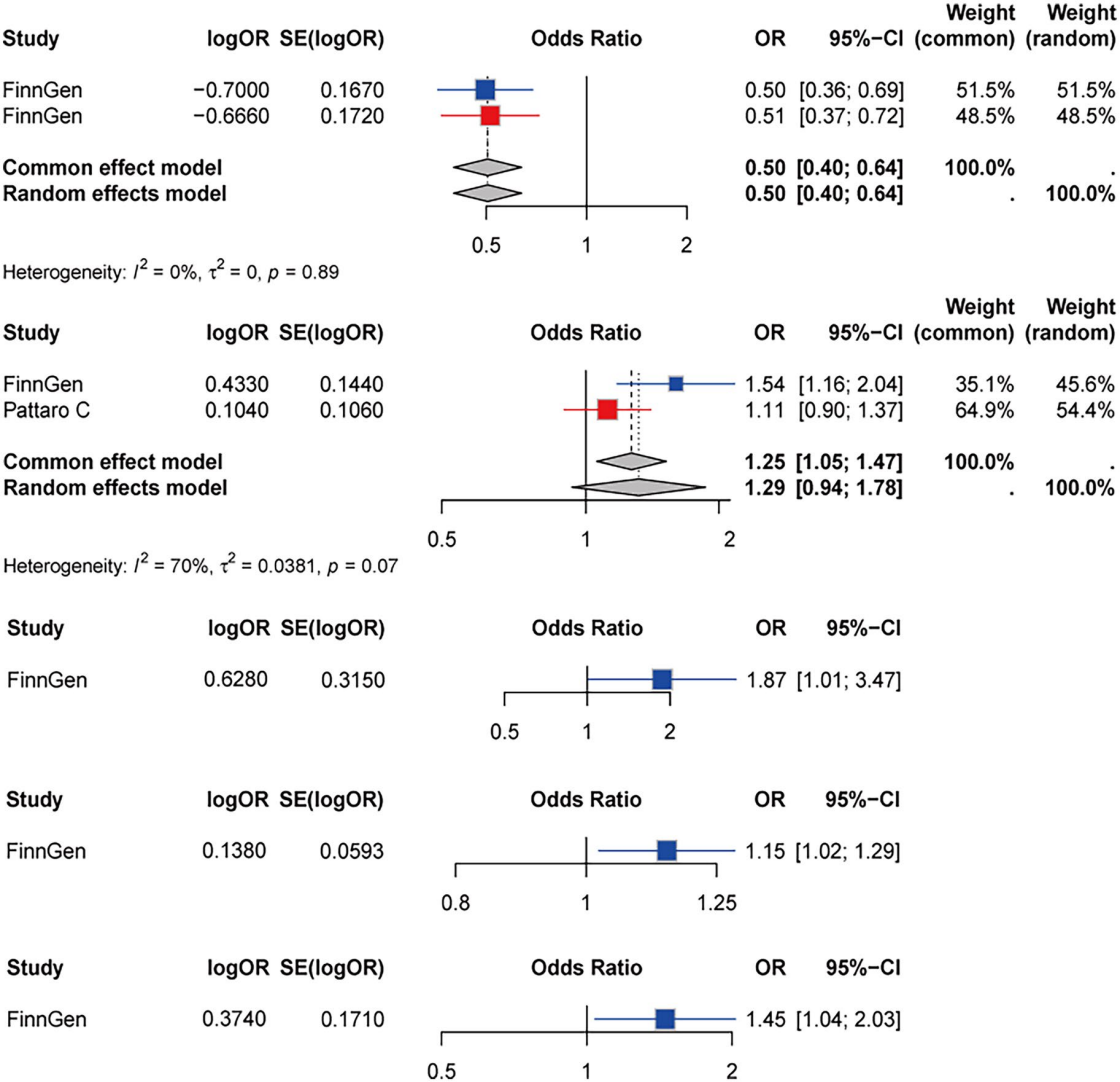
Associations of genetically predicted testicular hydrocele surgery with kidney diseases. From top to bottom, Benign neoplasm of kidney, Chronic kidney disease, Chronic tubulo-interstitial nephritis, Cystic kidney disease, and Glomerulonephritis (Inverse variance weighted method, *p* < 0.05). For combined results, *I*^2^ values < 25, 25–75, and > 75% were considered to indicate low, moderate, and high heterogeneity, respectively.

**Figure 3.**
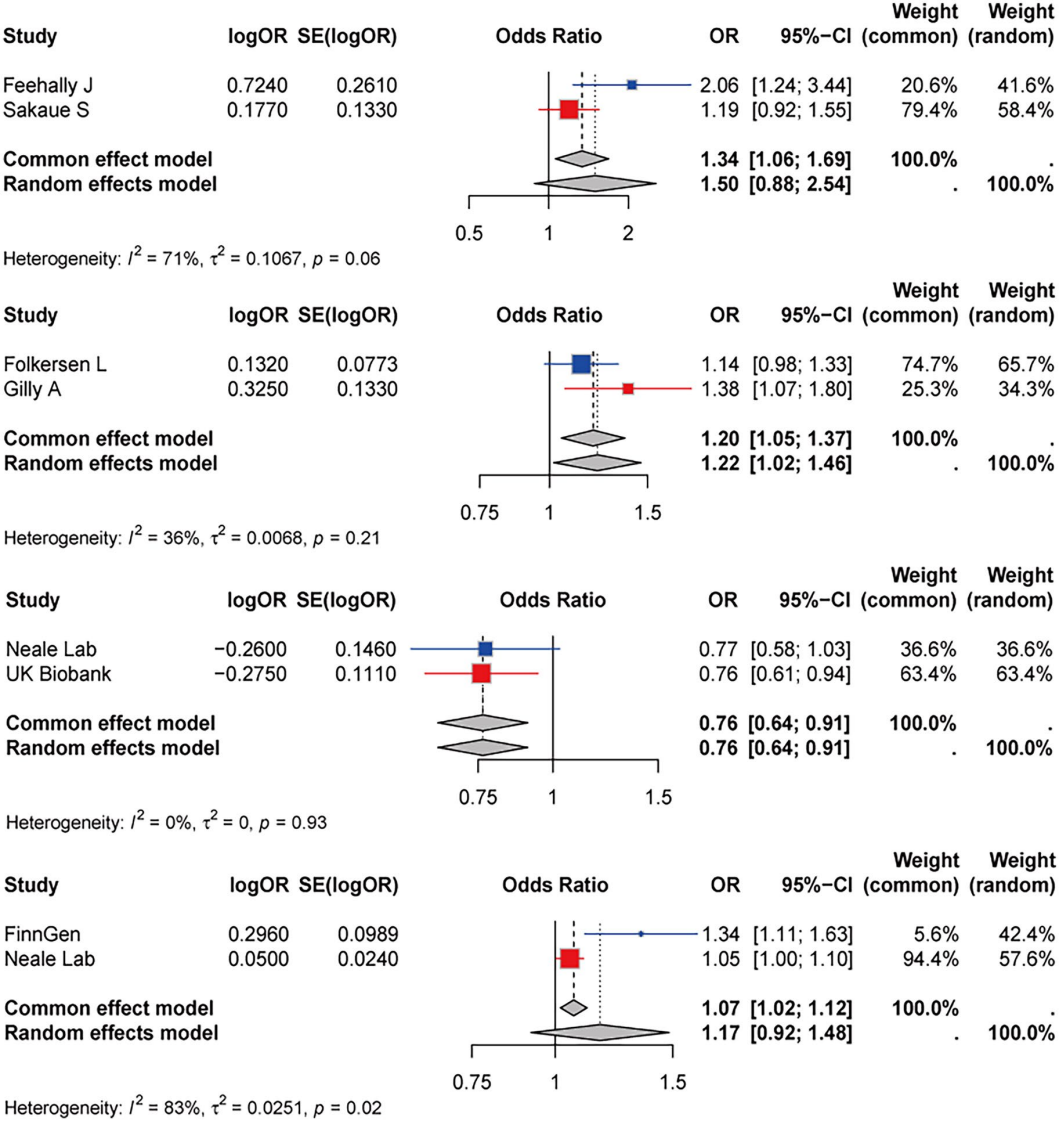
Associations of genetically predicted testicular hydrocele surgery with kidney diseases (continued from Figure 2). From top to bottom, IgA nephropathy, Kidney Injury Molecule levels, Kidney stone/ureter stone/bladder stone, and Renal failure. For combined results, *I*^2^ values < 25, 25–75, and > 75% were considered to indicate low, moderate, and high heterogeneity, respectively.

Utilizing testicular hydrocele surgery as the exposure, we extracted six SNPs as its IVs. The *F*-statistic for all IVs exceeded 1000, indicating no bias caused by weak instruments in our study (Table 2). In preliminary analysis and meta-analysis of all outcome data, genetic liability to undergoing testicular hydrocele surgery was associated with changed odds of nine kidney outcomes (Figures 2, 3).

For the three kidney diseases with only one source of outcome data available, a preliminary MR analysis was conducted. The results indicated that genetic liability to undergoing testicular hydrocele surgery was associated with higher odds of chronic tubulo-interstitial nephritis (OR: 1.87 [1.01–3.47]), cystic kidney disease (OR: 1.15 [1.02–1.29]), and glomerulonephritis (OR: 1.45 [1.04–2.03]).

For the six renal diseases with two or more sources of outcome data, we conducted replication analysis and meta-analysis. Choose the appropriate model based on the *I*^2^ statistic and the *P*-value of Cochrans’ *Q* test. The results demonstrated that genetic liability to undergoing testicular hydrocele surgery was associated with higher odds of chronic kidney disease (common effect model, OR: 1.25 [1.05–1.47]), IgA nephropathy (common effect model, OR: 1.34 [1.06–1.69]), and kidney injury molecule levels (common effect model, OR: 1.20 [1.05–1.37]). Additionally, lower odds was observed for benign neoplasm of kidney (common effect model, OR: 0.50 [0.40–0.64]) and kidney stone/ureter stone/bladder stone (common effect model, OR: 0.76 [0.64–0.91]). The relationship between testicular hydrocele surgery and renal failure was not significant after the combination (random effects models, OR: 1.17 [0.92–1.48]).

For preliminary analysis, after applying FDR correction, the associations with benign neoplasm of kidney, chronic kidney disease, renal failure, and IgA nephropathy remained statistically significant, while all other associations should be interpreted as exploratory (Supplementary Table S2). In the sensitivity analysis, heterogeneity was observed only in the causal relationship with IgA nephropathy as indicated by the Cochrans’ *Q* test (MR-Egger: *Q* = 10.14, *p* = 0.038; IVW: *Q* = 11.09, *p* = 0.050). However, MR-Egger regression results indicated that the pleiotropic effect was minimal (*p* = 0.575), suggesting that the presence of heterogeneity would not introduce significant bias into the MR estimates. No heterogeneity or pleiotropy was detected in the remaining analysis (Table 3). For continuous biomarker outcomes (e.g., KIM-1), MR effect estimates reflect standardized changes in biomarker levels; however, these should be interpreted cautiously, as unit definitions reflect the scaling used in the original GWAS rather than clinical measurement units.

**Table 3.** Results of sensitivity analysis.

Exposure	Outcome	Heterogeneity					
		MR Egger		IVW		Pleiotropy	
		*Q*	*P* value	*Q*	*P* value	Egger-intercept	*P* value
Testicular hydrocele surgery	Chronic tubulo-interstitial nephritis	2.27	0.69	2.39	0.79	−0.12	0.75
	Cystic kidney disease	5.96	0.20	7.81	0.17	−0.51	0.33
	Glomerulonephritis	4.83	0.31	4.83	0.44	0.00	1.00
	Chronic kidney disease (ebi-a-GCST003374)	1.14	0.77	2.32	0.68	0.12	0.36
	Chronic kidney disease (finn-b-N14_CHRONKIDNEYDIS)	2.84	0.59	2.91	0.71	−0.04	0.80
	IgA nephropathy (ebi-a-GCST90018866)	10.14	0.04	11.09	0.05	−0.07	0.58
	IgA nephropathy (ieu-a-1081)	0.01	0.91	0.17	0.92	−0.29	0.76
	kidney injury molecule levels (ebi-a-GCST90012041)	3.31	0.51	4.17	0.53	0.06	0.41
	kidney injury molecule levels (ebi-a-GCST90010147)	0.09	0.99	0.63	0.96	−0.19	0.51
	renal failure (finn-b-N14_RENFAIL)	1.02	0.91	2.06	0.84	−0.12	0.37
	Renal failure (ukb-a-573)	1.27	0.87	2.19	0.82	0.00	0.39
	Benign neoplasm of kidney (finn-b-CD2_BENIGN_KIDNEY_EXALLC)	0.44	0.98	0.44	0.99	0.01	0.98
	Benign neoplasm of kidney (finn-b-CD2_BENIGN_KIDNEY)	0.41	0.98	0.41	0.99	0.03	0.95
	kidney stone/ureter stone/bladder stone (ukb-b-8297)	2.68	0.61	3.08	0.69	0.00	0.56
	Kidney stone/ureter stone/bladder stone (ukb-a-72)	3.71	0.45	3.82	0.58	0.00	0.76
Testicular/scrotal surgery	Unspecified renal colic	0.35	0.84	0.40	0.94	0.00	0.85
	Gout due to impairment of renal function	2.66	0.75	2.66	0.85	0.04	0.95
	Malformations of renal pelvis and ureters	2.40	0.79	2.40	0.88	0.02	0.97
	Calculus of kidney and ureter (ukb-a-574)	1.88	0.87	2.14	0.91	0.00	0.63
	Calculus of kidney and ureter (finn-b-N14_CALCUKIDUR)	2.57	0.77	3.40	0.76	0.09	0.41
	Urinary tract infection or kidney infection (ebi-a-GCST90013940)	7.35	0.12	7.65	0.18	−0.02	0.70
	Urinary tract infection or kidney infection (ebi-a-GCST90013890)	7.35	0.12	7.65	0.18	−0.02	0.70
	Urinary tract infection or kidney infection (ukb-b-7150)	6.45	0.17	6.92	0.23	0.00	0.62
Male circumcision	Malformations of renal pelvis and ureters			0.02	0.89		
	Hypertensive heart/renal disease			0.47	0.49		
	Kidney volume			0.03	0.87		
	Malignant neoplasm of kidney			0.71	0.40		
	Cyst of kidney			0.01	0.91		
	Chronic renal failure			0.21	0.65		
	Benign neoplasm of kidney (finn-b-CD2_BENIGN_KIDNEY_EXALLC)			0.56	0.45		
	Benign neoplasm of kidney (finn-b-CD2_BENIGN_KIDNEY)			0.66	0.42		
Vasectomy	Pyelonephritis	13.15	0.83	13.17	0.87	0.00	0.90
	Malignant neoplasm of kidney (finn-b-C3_KIDNEY_NOTRENALPELVIS)	25.78	0.17	26.09	0.20	0.03	0.63
	Malignant neoplasm of kidney (ukb-b-1316)	0.46	0.98	0.90	0.97	0.00	0.54

### 3.2. Testicular or scrotal surgery and kidney diseases

**Figure 4.**
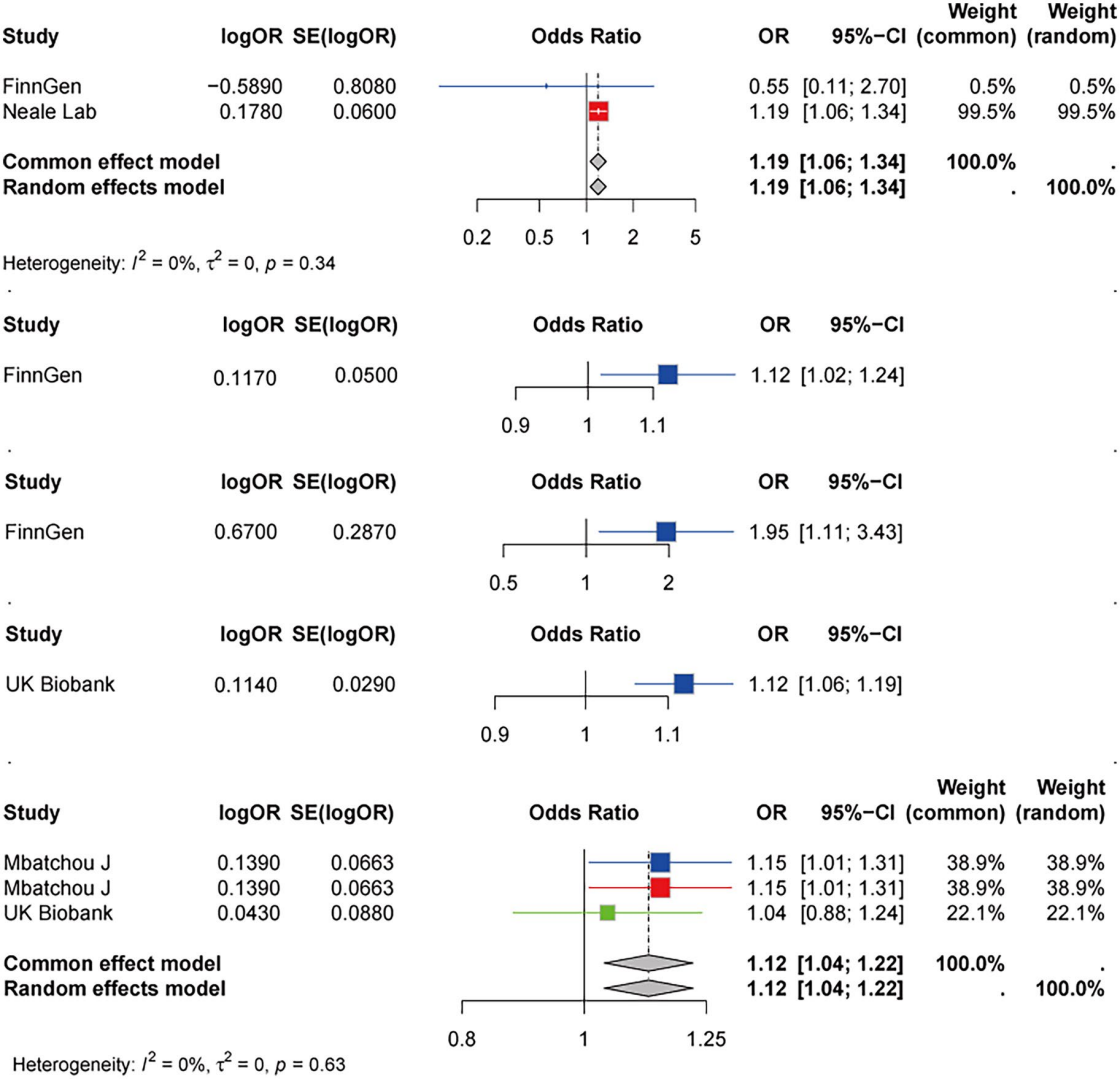
Associations of genetically predicted testicular or scrotal surgery with kidney diseases. From top to bottom, Calculus of kidney and ureter, Malformations of renal pelvis and ureters, Gout due to impairment of renal function, Unspecified renal colic, and Urinary tract infection or kidney infection. For combined results, *I*^2^ values < 25, 25–75, and > 75% were considered to indicate low, moderate, and high heterogeneity, respectively.

Utilizing testicular or scrotal surgery as the exposure, we extracted seven SNPs as IVs (Table 2). In preliminary analysis and meta-analysis of all outcome data, genetic liability to undergoing testicular or scrotal surgery was associated with changed odds of five kidney diseases (Figure 4).

For the three kidney diseases with only one source of outcome data available, the preliminary MR analysis indicated higher odds of unspecified renal colic (OR: 1.12 [1.06–1.19]), gout due to impairment of renal function (OR: 1.12 [1.02–1.24]), and malformations of renal pelvis and ureters (OR: 1.95 [1.11–3.43]).

For the two renal diseases with two or more sources of outcome data, replication analysis and meta-analysis demonstrated higher odds of calculus of kidney and ureter (common effect model, OR: 1.19 [1.06–1.34]) and urinary tract infection or kidney infection (common effect model, OR: 1.12 [1.04–1.22]).

For preliminary analysis, after applying FDR correction, the association between genetic liability to testicular or scrotal surgery and unspecified renal colic remained statistically significant, whereas the other associations did not survive multiple-testing correction and should be interpreted as exploratory (Supplementary Table S2). In the sensitivity analysis, no heterogeneity or pleiotropy was detected. This indicates that our findings are robust and reliable, mitigating the risk of bias and endogeneity issues (Table 3).

### 3.3. Male circumcision and kidney diseases

**Figure 5.**
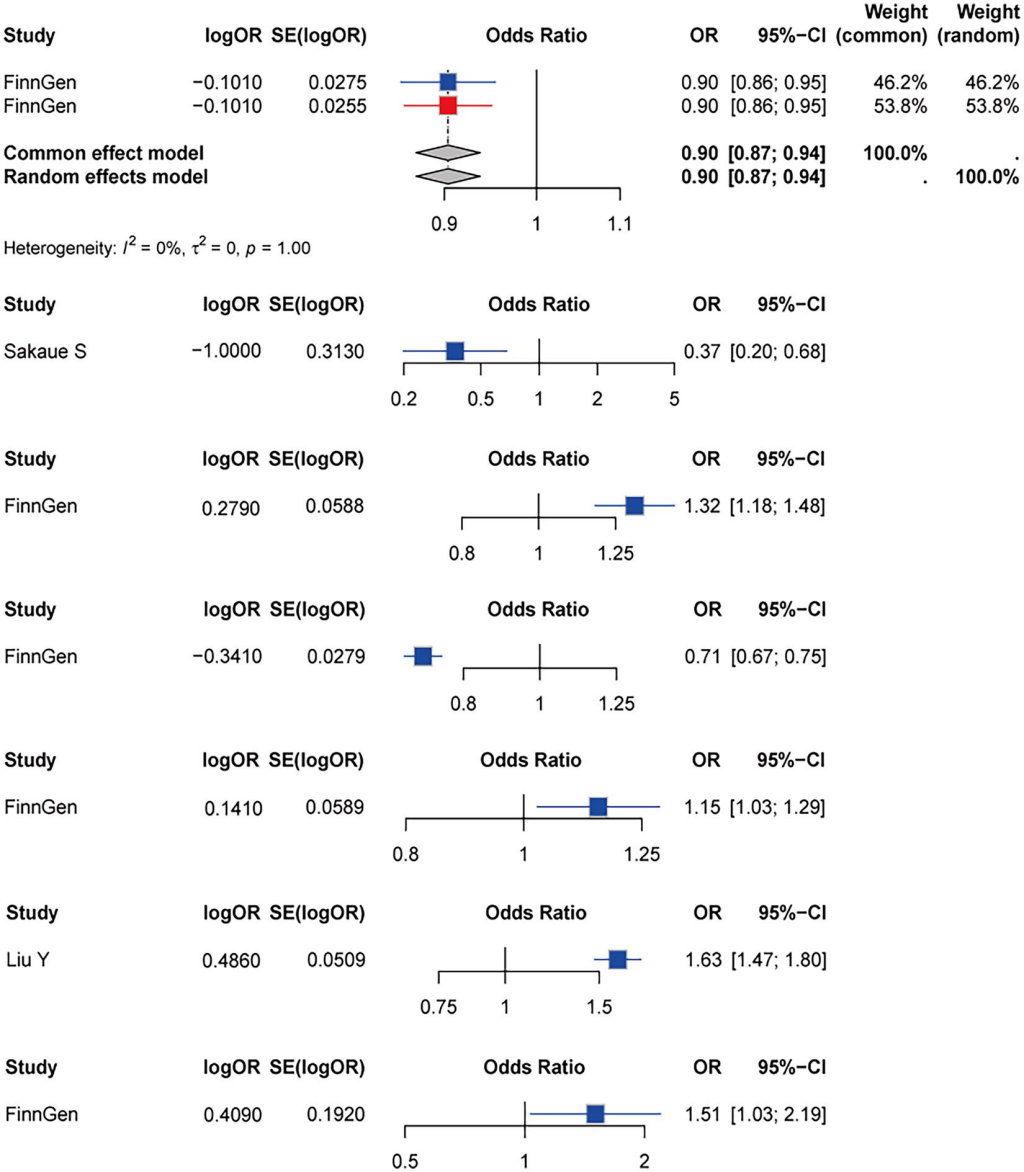
Associations of genetically predicted male circumcision with kidney diseases. From top to bottom, Benign neoplasm of kidney, Chronic renal failure, Malformations of renal pelvis and ureters, Cyst of kidney, Hypertensive heart/renal disease, Kidney volume, and Malignant neoplasm of kidney. For combined results, *I*^2^ values < 25, 25–75, and > 75% were considered to indicate low, moderate, and high heterogeneity, respectively.

Utilizing male circumcision as the exposure, we extracted two SNPs as IVs (Table 2). In preliminary analysis and meta-analysis of all outcome data, genetic liability to undergoing male circumcision showed associations with changed odds of seven kidney diseases (Figure 5).

For the six kidney diseases with only one source of outcome data available, the preliminary MR analysis indicated higher odds of malformations of renal pelvis and ureters (OR: 1.32 [1.18–1.48]), hypertensive heart/renal disease (OR: 1.15 [1.03–1.29]), kidney volume (OR: 1.63 [1.47–1.80]), and malignant neoplasm of the kidney (OR: 1.51 [1.03–2.19]). Additionally, lower odds were observed for cyst of kidney (OR: 0.71 [0.67–0.75]) and chronic renal failure (OR: 0.37 [0.20–0.68]).

For benign neoplasm of kidney, replication analysis and meta-analysis demonstrated that male circumcision was associated with lower odds (common effect model, OR: 0.90 [0.87–0.94]).

For preliminary analysis, after applying FDR correction, the associations with benign neoplasm of kidney, chronic renal failure, malformations of renal pelvis and ureters, cyst of kidney, and kidney volume remained statistically significant, while all other associations should be interpreted as exploratory (Supplementary Table S2). In the sensitivity analysis, no heterogeneity was detected. Additionally, the MR-Egger regression for detecting pleiotropy was not applicable due to the lack of IVs (Table 3).

### 3.4. Vasectomy and kidney diseases

**Figure 6.**
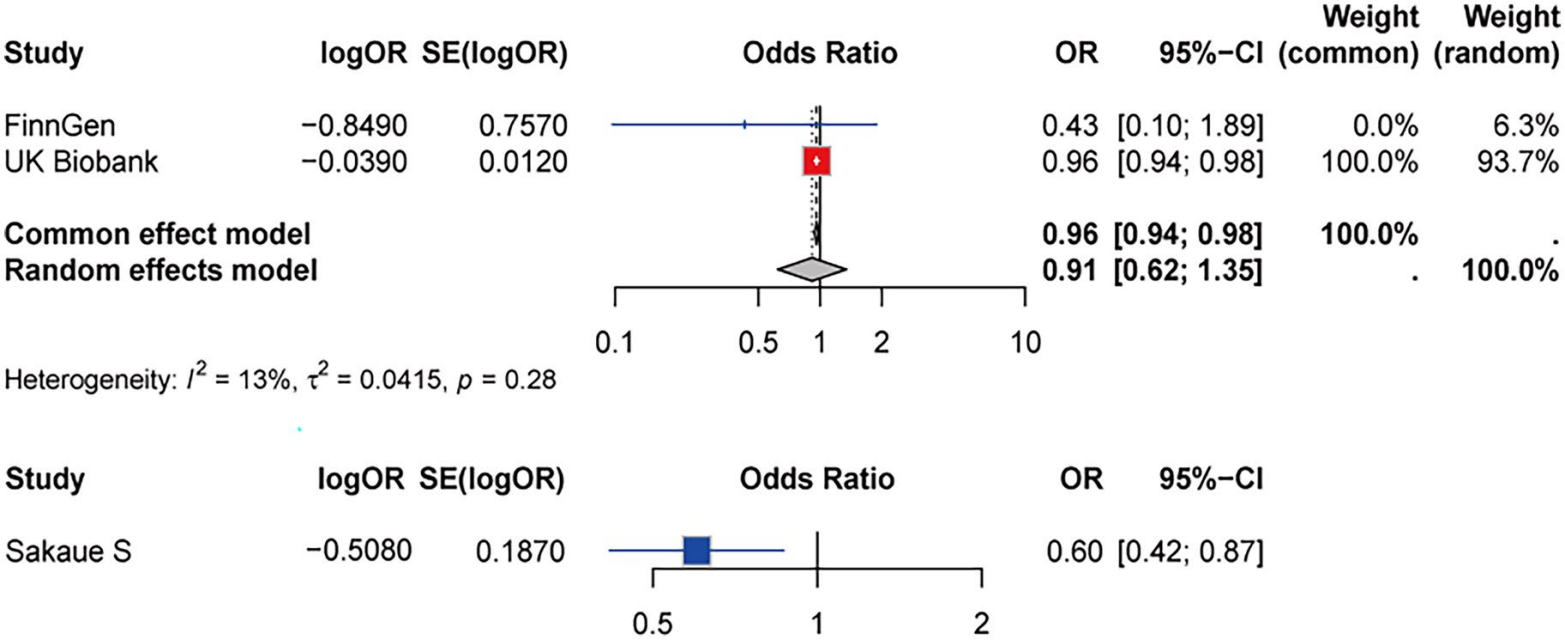
Associations of genetically predicted vasectomy with kidney diseases. From top to bottom, Benign neoplasm of kidney, Malignant neoplasm of kidney and Pyelonephritis. For combined results, *I*^2^ values < 25, 25–75, and > 75% were considered to indicate low, moderate, and high heterogeneity, respectively.

Utilizing vasectomy as the exposure, we extracted twenty-two SNPs as IVs (Table 2). In preliminary analysis and meta-analysis of all outcome data, genetic liability to undergoing vasectomy was associated with lower odds of two kidney diseases: pyelonephritis (OR: 0.60 [0.42–0.87]) and malignant neoplasm of kidney (Common effect model, OR: 0.96 [0.94–0.98]) (Figure 6). Given the very small effect size for malignant kidney neoplasm (OR ≈ 1), this association should be interpreted cautiously, as it may reflect residual confounding or statistical noise.

For preliminary analysis, after applying FDR correction, the association between genetic liability to vasectomy and malignant neoplasm of the kidney remained statistically significant, whereas the other associations did not survive multiple-testing correction and should be interpreted as exploratory (Supplementary Table S2). In the sensitivity analysis, no heterogeneity or pleiotropy was detected, indicating that our findings are robust and reliable (Table 3).

### 3.5. Mediation analysis

**Figure 7.**
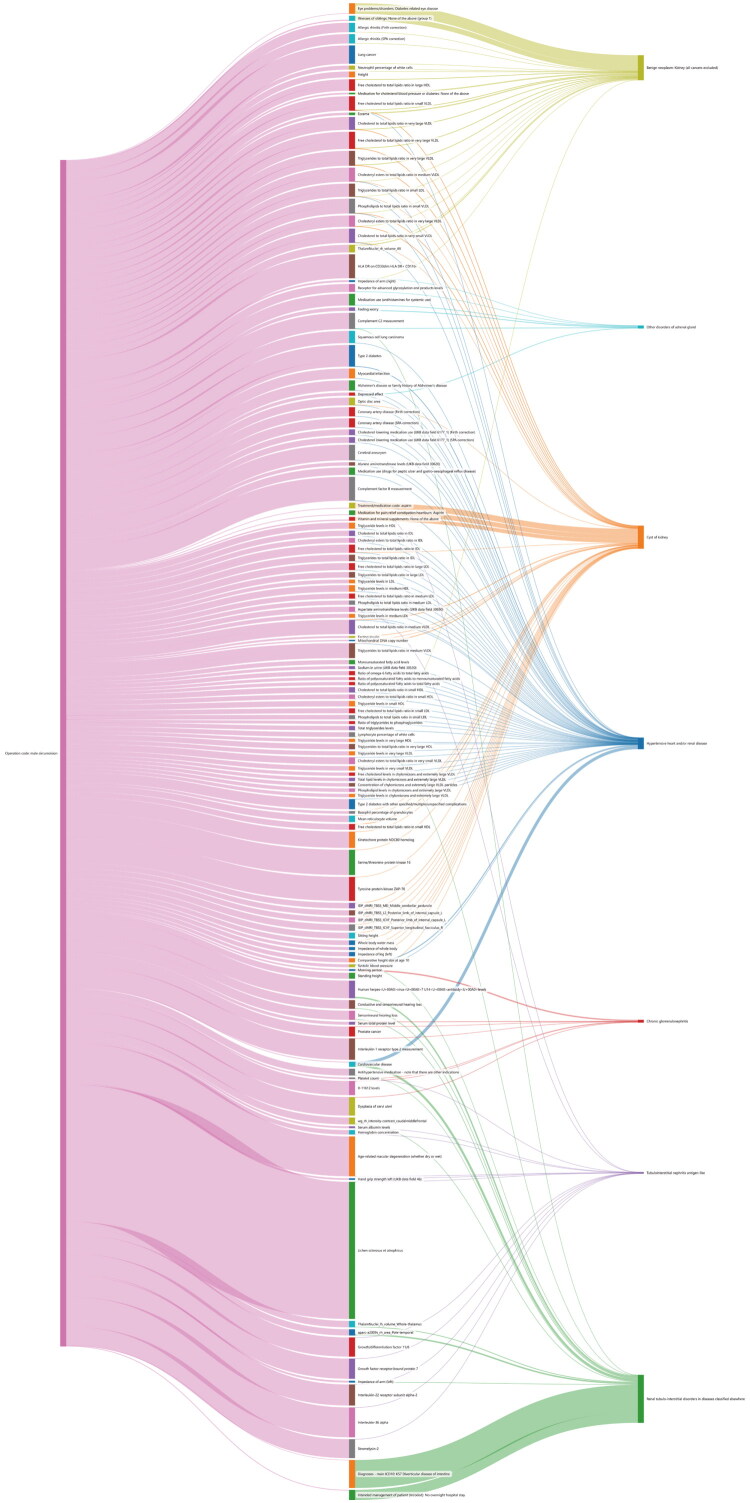
Sankey diagram of mediated analysis of kidney disease by male reproductive system surgery. Mediators were selected based on their biological relevance to kidney disease and their potential role in the mechanisms linking male reproductive system surgeries to kidney diseases. These included: (1) Clinical indicators, such as blood pressure, lipid profiles, and kidney injury biomarkers. (2) Inflammatory and immune markers, including cytokines and HLA markers. (3) Metabolic factors, such as fasting insulin and serum albumin levels.

Using a two-step Mendelian randomization framework, we explored potential mediating pathways linking genetic liability to undergoing circumcision with kidney-related outcomes (other surgeries were excluded due to lack of significant association). Because all genome-wide significant variants for circumcision lie within the HLA region, the exposure instrument reflects an immune-related genetic liability rather than biological effects of the circumcision procedure itself. Therefore, the mediation findings should be interpreted as exploratory signals arising from the immunogenetic architecture underlying circumcision liability, rather than as mechanistic pathways directly caused by the surgical act (Figure 7).

In the first step, genetic liability to circumcision showed associations with several clinically relevant traits, including hemoglobin concentration (*β* = 6.57, *p* = 0.0001), neutrophil percentage (*β* = 5.93, *p* = 0.0004), and fasting insulin (*β* = 2.91, *p* = 0.0141). These traits are not HLA markers themselves; instead, they likely reflect downstream hematological, immune, and metabolic consequences of the broader genetic background associated with circumcision liability (Supplementary Table S3).

In the second step, these mediators were further associated with kidney-related outcomes. Hemoglobin concentration was linked to tubulointerstitial nephritis antigen-like levels (*β* = 0.18, *p* = 0.0189), neutrophil percentage to benign kidney neoplasms (*β* = 0.37, *p* = 0.0484), and fasting insulin to kidney cysts (*β* = 0.98, *p* = 0.0351). Additionally, the cholesterol-to-total-lipids ratio in IDL was associated with hypertensive heart and/or renal disease (*β* = −0.14, *p* = 0.0188). These findings suggest that immune and metabolic traits may act as intermediate pathways connecting the genetic architecture underlying circumcision liability with renal phenotypes (Supplementary Table S3).

Sensitivity analyses revealed no major violations of MR assumptions; however, because circumcision instruments reside entirely within the highly pleiotropic HLA locus, these mediation pathways should be viewed as hypothesis-generating and interpreted cautiously. Further studies with non-HLA instruments or mechanistic data are required to confirm these indirect associations.

## 4. Discussion

In this study, we present the first comprehensive MR analysis to investigate whether genetic liability to undergoing different male reproductive system surgeries is associated with a wide range of kidney diseases. By integrating meta-analyses and exploratory mediation analyses, we evaluated how predisposition to the underlying indications for these procedures may relate to diverse renal outcomes. Our analyses identified several statistically robust and exploratory associations, providing new insights into potential shared biological pathways that may link these underlying predispositions with renal phenotypes. These findings underscore the importance of considering kidney health in individuals presenting with conditions that commonly lead to male reproductive surgeries, while also highlighting the need for further mechanistic and epidemiological studies to clarify the pathways involved.

A key conceptual consideration in this study is the nature of the exposure being instrumented. The SNPs used in our analyses do not capture the biological or postoperative effects of male reproductive system surgeries. Instead, they instrument genetic liability to being coded for a surgery, which reflects predisposition to the underlying conditions, anatomical features, developmental pathways, behaviors, or cultural factors that influence whether an individual undergoes a given procedure. The occurrence of surgery is also shaped by non-genetic factors such as healthcare access, medical decision-making, provider practices, and sociocultural norms, none of which are directly represented by genetic instruments.

This distinction carries important implications for the MR assumptions, particularly the exclusion–restriction assumption, which requires that genetic variants influence kidney outcomes only through the liability leading to the surgery phenotype. Under this framework, our estimates reflect the consequences of the underlying indications for surgery, rather than the surgical procedures themselves. Thus, findings should not be interpreted as postoperative biological effects, but rather as associations arising from shared genetic predispositions that increase the likelihood of both surgery-coded phenotypes and specific renal outcomes. Recognizing this distinction is essential for accurate interpretation of the MR results presented in this study.

Testicular hydrocele surgery is the primary mode of treatment for hydrocele, a condition affecting males across all age groups, with an estimated prevalence of 1% in adult men and 0.7%–4.7% in male infants [1,24]. In England, approximately 0.15% of boys undergo testicular hydrocele surgery each year, corresponding to about 2,878 procedures annually [1]. While postoperative complications such as hematoma, pain, and infection have been well characterized [25–27], little is known about the broader health profiles of individuals who are genetically predisposed to conditions that lead to hydrocele surgery. In our MR analysis, genetic liability to undergoing testicular hydrocele surgery was associated with a heterogeneous pattern of renal outcomes, including higher risks of tubulo-interstitial nephritis, glomerulonephritis, IgA nephropathy, and cystic kidney disease, alongside lower risks of benign kidney tumors and urinary stones. These associations likely reflect shared biological pathways underlying hydrocele susceptibility and renal phenotypes, rather than consequences of the surgical procedure itself. The male reproductive and urinary systems share developmental origins, anatomical proximity, and vascular–lymphatic connections, which may explain why individuals genetically predisposed to hydrocele may also exhibit susceptibility to immune, inflammatory, or structural processes relevant to kidney function.

Testicular or scrotal surgery is a collective term for various procedures, including orchiectomy, testicular fixation, hydrocele surgery, and varicocele repair. Most of these surgeries are associated with postoperative pain, bleeding, and wound infections, and some can lead to serious complications [7,28]. For example, a national cohort study involving 14,715 samples showed that orchiectomy was associated with higher rates of cardiovascular ischemic events following prostate cancer [29]. Another large cohort study involving 69,292 samples suggested that orchiectomy may increase the risk of acute kidney injury in prostate cancer patients [12]. In our MR analyses, genetic liability to undergoing testicular or scrotal surgery was associated with increased risks of renal colic, gout secondary to impaired kidney function, urinary stones, and kidney infections. These findings do not imply causal effects of the surgical procedures themselves; rather, they suggest that individuals who are genetically predisposed to the underlying conditions necessitating testicular or scrotal surgery may also carry shared biological, metabolic, or anatomical susceptibilities that influence renal–urinary outcomes. The interconnected development and close anatomical relationship of the testicular, scrotal, and urinary systems provide a plausible framework for these shared susceptibilities. This multifaceted genetic and physiological overlap may underlie the observed heterogeneity in renal traits among individuals with a higher genetic propensity for testicular or scrotal surgical indications.

Male circumcision is one of the most common surgical procedures globally, with an estimated 30% of the male population having undergone the procedure [30]. Its short-term postoperative complications—such as bleeding, infection, adhesions, and urethral stricture—have been well documented in large meta-analyses [8]. Although only limited observational evidence has examined kidney-related consequences, occasional case reports have described obstructive uropathy and acute kidney injury following circumcision [31–33]. In our MR analyses, however, the associations do not reflect biological effects of the circumcision procedure. Instead, because all genome-wide significant variants for circumcision reside within the HLA region, the MR estimates capture an immune-related genetic liability to being coded for circumcision, which may reflect cultural, behavioral, developmental, or immunogenetic factors linked to circumcision status. This genetic liability was associated with several renal phenotypes in both directions—including higher risks of congenital obstructive defects, hypertensive heart/renal disease, and malignant kidney neoplasm, as well as lower risks of kidney cysts and chronic renal failure.

Vasectomy is a widely accepted method of male sterilization worldwide, valued for its simplicity, safety, effectiveness, and cost-efficiency [4]. By 2006, approximately 43 million men had undergone the procedure [34]. While short-term complications such as hematoma, pain, and infection have been documented [4], long-term observational studies generally report no increased risks of autoimmune disorders [35], cardiovascular diseases [36], prostate cancer [37], or sexual dysfunction [38]. In our MR analyses, genetic liability to undergoing vasectomy, which may reflect behavioral, reproductive, or sociodemographic characteristics associated with choosing vasectomy, was not associated with elevated risks of kidney diseases. Instead, modest inverse associations were observed for pyelonephritis and malignant kidney neoplasm. These associations should not be interpreted as protective biological effects of the vasectomy procedure itself; rather, they may reflect underlying genetic or lifestyle factors correlated with the likelihood of undergoing vasectomy. Overall, the findings suggest that the genetic predisposition to characteristics or life circumstances that lead individuals to undergo vasectomy does not confer increased renal risk. While the observed inverse associations warrant further investigation, they should be viewed as exploratory given the small effect sizes and the potential influence of correlated behavioral or demographic traits.

Our study possesses several notable strengths. First, to the best of our knowledge, this is the first Mendelian randomization analysis to systematically examine the relationships between genetic liability to undergoing male reproductive system surgeries—reflecting their underlying indications—and a broad spectrum of kidney diseases. Although research on these relationships remains scarce, the anatomical and developmental interconnectedness of the male reproductive and urinary systems provides a rationale for investigating shared genetic pathways that may predispose individuals to both surgical indications and renal phenotypes. Second, by performing replication analyses and meta-analyses across multiple large-scale GWAS datasets, we substantially improved statistical power and enhanced the robustness and generalizability of the observed associations. This multi-cohort approach reduces the influence of dataset-specific biases and strengthens confidence in the consistency of the findings. Finally, we incorporated an exploratory mediation analysis to investigate whether immunological, hematologic, or metabolic traits might partly explain the associations between genetic liability to circumcision and selected kidney outcomes. Although these findings should be interpreted cautiously due to the pleiotropic nature of HLA-region instruments, they provide preliminary insights into potential biological pathways that warrant further mechanistic and clinical investigation.

Despite its strengths, this study has several important limitations. First, Mendelian randomization estimates reflect genetic liability to undergoing male reproductive system surgeries—driven by the underlying congenital, developmental, cultural, or behavioral indications for surgery—rather than the biological effects of the surgical procedures themselves. This gene–environment nonequivalence limits direct comparison with short-term observational studies and means that the associations observed here should not be interpreted as postoperative physiological consequences. Second, although large publicly available GWAS datasets were used, replication was not possible for certain kidney outcomes because only a single dataset existed, potentially reducing the robustness of these specific associations. Third, while we explored potential intermediary traits, the biological interpretation remains exploratory, particularly because small effect sizes (e.g., ORs close to 1.00) may reflect modest biological influence, correlated behaviors, or random variation rather than meaningful clinical associations. Fourth, as all exposure GWAS and most outcome GWAS were conducted among individuals of European ancestry, our findings may not generalize to other populations. This is especially relevant for circumcision, where genetic liability to circumcision coding may capture cultural, religious, socioeconomic, or healthcare-access patterns rather than biological predisposition, and where MR cannot distinguish infant circumcision from adult circumcision. Finally, we did not exclude variants located within the HLA/MHC region. This was necessary because the only genome-wide significant instruments for circumcision—and several instruments for other exposures—were located in this region. However, the HLA locus is known to exhibit extensive immune-related pleiotropy. Although sensitivity analyses did not reveal substantial horizontal pleiotropy, residual pleiotropic effects cannot be ruled out, and circumcision-related findings in particular should be interpreted with caution. Future studies using non-MHC instruments or individual-level data may help clarify these relationships.

## 5. Conclusion

This study provides suggestive evidence that genetic liability to undergoing different male reproductive surgeries—reflecting the underlying biological, developmental, or behavioral indications for these procedures—is associated with distinct patterns of kidney disease risk. These associations improve our understanding of the shared genetic and physiological architecture linking reproductive and renal traits, while emphasizing the complexity of these relationships. Importantly, this study also provides the first MR-based evidence that immune and metabolic traits may act as intermediate pathways linking the underlying indications for these surgeries with kidney outcomes. Given the nature of the instruments and the modest effect sizes observed, the findings should be interpreted as exploratory, rather than indicative of clinical effects of the surgical procedures themselves. Nonetheless, the results highlight areas where future mechanistic, epidemiological, and genetic studies may help clarify the pathways underlying these associations. Such work could ultimately support the development of a more nuanced understanding of renal risk in populations predisposed to the underlying conditions that lead to these surgeries.

## Supplementary Material

Supplemental Material

Supplemental Material

Supplemental Material

## Data Availability

The data that support the findings of this study, including Supplementary Table S1–S3, are openly available in the Zenodo repository at https://doi.org/10.5281/zenodo.19652432.

## Abbreviations

MR: Mendelian randomization
IVW: Inverse variance weighted
GWAS: Genome-wide association studiy
SNPs: Single nucleotide polymorphisms
CKD: Chronic kidney disease
ESRD: End-stage renal disease
AKI: Acute kidney injury
OR: Odds ratio
CI: Confidence interval
LD: Linkage disequilibrium
Ivs: Instrumental variables
